# Tandem mass tag-based quantitative proteomic analysis of lycorine treatment in highly pathogenic avian influenza H5N1 virus infection

**DOI:** 10.7717/peerj.7697

**Published:** 2019-10-02

**Authors:** Li Yang, Jia Hao Zhang, Xiao Li Zhang, Guang Jie Lao, Guan Ming Su, Lei Wang, Yao Lan Li, Wen Cai Ye, Jun He

**Affiliations:** 1 Guangdong Province Key Laboratory of Pharmacodynamic Constituents of TCM and New Drugs Research, Jinan University, Guangzhou, China; 2 College of Life Science and Technology, Jinan University, Guangzhou, China; 3 College of Veterinary Medicine, South China Agricultural University, Guangzhou, China; 4 Institute of Laboratory Animal Science, Jinan University, Guangzhou, China

**Keywords:** Highly pathogenic avian influenza virus, Multiplex tandem mass tag, Lycorine, Nuclear-cytoplasmic transport, Nuclear pore complex protein 93

## Abstract

Highly pathogenic H5N1 influenza viruses (HPAIV) cause rapid systemic illness and death in susceptible animals, leading to a disease with high morbidity and mortality rates. Although vaccines and drugs are the best solution to prevent this threat, a more effective treatment for H5 strains of influenza has yet to be developed. Therefore, the development of therapeutics/drugs that combat H5N1 influenza virus infection is becoming increasingly important. Lycorine, the major component of Amaryllidaceae alkaloids, exhibits better protective effects against A/CK/GD/178/04 (H5N1) (GD178) viruses than the commercial neuraminidase (NA) inhibitor oseltamivir in our prior study. Lycorine demonstrates outstanding antiviral activity because of its inhibitory activity against the export of viral ribonucleoprotein complexes (vRNPs) from the nucleus. However, how lycorine affects the proteome of AIV infected cells is unknown. Therefore, we performed a comparative proteomic analysis to identify changes in protein expression in AIV-infected Madin-Darby Canine Kidney cells treated with lycorine. Three groups were designed: mock infection group (M), virus infection group (V), and virus infection and lycorine-treated after virus infection group (L). The multiplexed tandem mass tag (TMT) approach was employed to analyze protein level in this study. In total, 5,786 proteins were identified from the three groups of cells by using TMT proteomic analysis. In the V/M group, 1,101 proteins were identified, of which 340 differentially expressed proteins (DEPs) were determined during HPAIV infection; among the 1,059 proteins identified from the lycorine-treated group, 258 proteins presented significant change. Here, 71 proteins showed significant upregulation or downregulation of expression in the virus-infected/mock and virus-infected/lycorine-treated comparisons, and the proteins in each fraction were functionally classified further. Interestingly, lycorine treatment decreased the levels of the nuclear pore complex protein 93 (Nup93, E2RSV7), which is associated with nuclear–cytoplasmic transport. In addition, Western blot experiments confirmed that the expression of Nup93 was significantly downregulated in lycorine treatment but induced after viral infection. Our results may provide new insights into how lycorine may trap vRNPs in the nucleus and suggest new potential therapeutic targets for influenza virus.

## Introduction

Highly pathogenic influenza A viruses resulting from routine seasonal epidemics and global pandemics continue to circulate in nature. It is an extremely contagious and aggressive disease that causes rapid systemic illness and death in susceptible birds. Moreover, certain strains, such as H5N1 subtypes, are capable of cross-species transmission and thus can infect humans with high morbidity and mortality (Sun et al., 2015). The new subtypes have emerged through accumulation and antigenic shift (Zhu, Wang & Wang, 2017), which may result in rare influenza A virus pandemics. Resistance and timeliness to clinical antiviral therapy are the major problems in curing these infections (Blanton et al., 2017; Hu et al., 2017). M2 inhibitors, such as amantadine and ramantadine, cannot protect the host from virus infection because of its amino acid mutation (Wang et al., 2013). To date, oseltamivir is the only commercial drug effective in human, where it inhibits NA activity. However, drug-resistant strains have emerged because of one single amino acid residue substitution (H274Y in N1) (Yusuf et al., 2016). Therefore, novel influenza inhibitors must be developed.

Virus–host interactions involve a complex interplay of host cellular and viral networks. The discovery of host factors that may regulate viral replication is a promising approach. In this respect, genomic and proteomic studies have provided abundant information on host genes and proteins associated with viral infection and pathogenesis (Wang et al., 2014; Zhang et al., 2018). The mode of RNP exit of influenza viruses from the nucleus complements active Crm1-dependent export mechanisms via the nuclear pore complex (NPC) and ensures the efficient production of infectious virus progeny (Muhlbauer et al., 2015). Some of the host factors involved in viral replication and/or pathogenesis could be targeted for the treatment of AIV infection without obvious side effects.

Traditional Chinese medicines (TCMs) contain effective components that deal with different types of diseases, such as malaria and influenza A virus (Crunkhorn, 2016; Nonaka et al., 2018). Lycorine is a TCM that exhibits acetyl-cholinesterase-inhibitory and butyryl-cholinesterase-inhibitory activities (Wang et al., 2012). In Russia, it is clinically used as an expectorant to treat chronic and acute inflammatory processes in lungs and bronchial diseases (Henry et al., 2016). Over the past years, lycorine has attracted great research interest owing to its superlative biological potential and pharmacological actions, including antiangiogenic, antiviral, antibacterial, antimalarial, anti-parasite, antioxidant, hepatoprotective, analgesic, anti-inflammatory activities, and inhibition of ascorbic acid synthesis (Kim et al., 2008; Lamoral-Theys et al., 2009; Cedron et al., 2010; Giordani et al., 2011; Citoglu et al., 2012; Ye et al., 2012; Wang et al., 2014; Cembrowska-Lech & Kepczynski, 2016; Bendaif et al., 2018). Different drugs may affect different molecular pathways that may be affected within disease models. For instance, several studies have shown that lycorine induces apoptosis to suppress the propagation of carcinoma cells (Li et al., 2007; Yu et al., 2017). Other studies have demonstrated that its antitumor effect is through inhibiting inflammatory factors and suppressing p38 and STAT activation (Kang et al., 2012). Although lycorine exhibits a wide range of biological activities, the mechanism underlying its effects remains unclear.

In our previous study, the efficacy and inhibitory effects of lycorine (EC_90_ = 0.52 µM) were determined in Madin-Darby Canine Kidney (MDCK) cells. Results showed that lycorine can completely prevent H5N1 infection as indicated by the absence of any cytopathic effect (He et al., 2013). Unlike the mechanism of oseltamivir, the vRNPs are retained in the nucleus by lycorine treatment instead of directly targeting viral proteins and RNA polymerase activity (He et al., 2013). These results strongly suggest the potential of lycorine as an antiviral agent for H5N1 strain. However, the molecular mechanism of the antiviral/inhibitor action of lycorine remains unknown. Therefore, we performed a comparative proteomic analysis to determine the effects of lycorine at the protein level in GD178-infected MDCK cells to understand its mode of action.

## Materials & Methods

### Virus, cells, and lycorine

**Figure 1:**
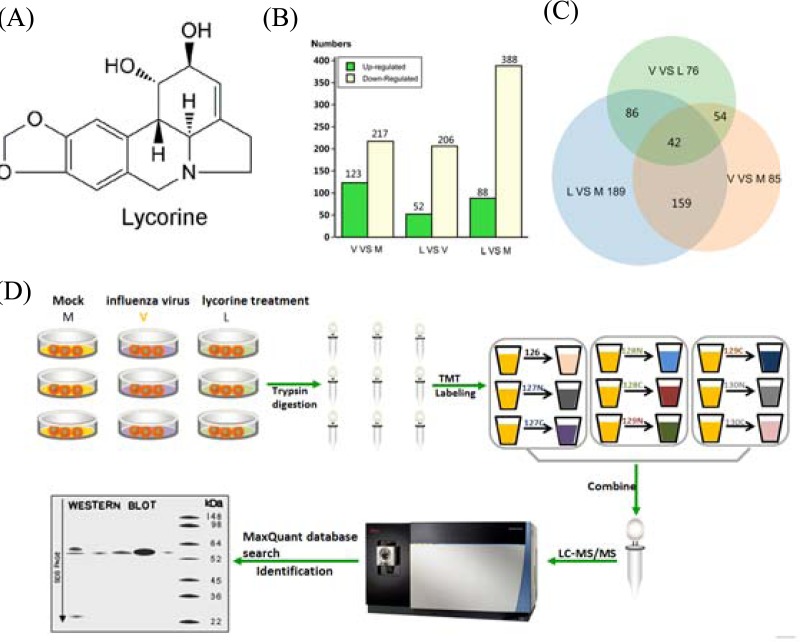
Schematic of experimental workflow and Venn diagram in V/M, L/V, and L/M. (A) Structure of lycorine. (B) Barchart of the number of unique differentially expressed proteins in different comparative groups. The *x*-axis indicates the comparisons between each two groups. The left *y*-axis shows the number of differentially expressed proteins. (C) Venn diagrams of DEPs in different comparative groups. Venn diagram of comparisons among V-VS-M, V-VS-L and L-VS-M. (D) Cells from each group were harvested, underwent trypsin digestion, and then labeled by tandem mass tag (TMT)-10 plex, and high-pH reverse-phase liquid chromatography was used to fractionate the pooled TMT-labeled peptide mixtures before nano liquid chromatography-mass spectroscopy tandem (LC-MS/MS) analysis. All raw files were searched together using Proteome Discover 2.1. The quantification data matrix was further used for statistical trend analysis and visualization by Trelliscope. Finally, Western blot experiments were conducted for trend verifications.

GD178 (**N0. AY737296-737300**) and MDCK cells were obtained from the Key Laboratory of Veterinary Vaccine Innovation of the Ministry of Agriculture, P. R. China. The MDCK cells were cultured in Dulbecco’s modified Eagle’s medium (DMEM, Invitrogen) containing 10% (v/v) fetal bovine serum (FBS, Gibco). A plaque assay was used to determine viral activity in MDCK cells (He et al., 2013). Three separate biological cell cultures were made for the tandem mass tag (TMT) proteomic experiments. Lycorine (Fig. 1A) was obtained as previously described (Wang et al., 2009). All experiments involving live H5N1 influenza virus were carried out in biosafety level-3 facilities.

### TMT assay design and protein preparation

Three groups were designed: (1) M, mock group (control); (2) V, virus-infected group. They were then inoculated at 3 multiplicity of infection (MOI) for 1 h (V), removed, and inoculated with DMEM containing 10% FBS until 12 h post-infection (h.p.i.); and (3) L, lycorine-treated post-viral infected group. After 1 h adsorption of 3 MOI GD178, lycorine was added at 0.52 µM for 12 h.p.i. For each sample, the proteins were diluted in 300 µL of SDT buffer (containing 4% SDS, 100 mM Tris-HCl, and 100 mM DTT, pH 8.0) following a standardized protocol (Wisniewski et al., 2009), homogenized, heated at 100 °C for 3 min, and then centrifuged at 14,000 × g for 30 min at room temperature. Each sample (one µL) was taken and quantified using the bicinchoninic acid method. The remaining lysate was frozen at −80 °C until use.

### Trypsin digestion

Protein digestion was conducted using the FASP procedure (Wisniewski et al., 2009). In brief, 300 mg of proteins were loaded onto an ultrafiltration filter (30 kDa cutoff; Sartorius, Goettingen, Germany) containing 200 µL of UA buffer (8 M urea, 150 mM Tris-HCl, pH 8.0), centrifuged at 14,000× g for 30 min, and then washed with 200 µL of UA buffer. Then, 100 µL of 50 mM iodoacetamide in UA buffer was subsequently added to the filter to block reduced cysteine residues. The samples were incubated for 30 min at room temperature in the dark and then centrifuged at 14,000× g for 30 min. The filters were washed three times with 100 µL of UA buffer and then centrifuged at 14,000× g for 30 min after each washing step. Next, 100 µL of dissolution buffer (Applied Biosystems, Foster City, CA, USA) was added to each filter and then centrifuged at 14,000× g for 30 min, which was repeated three times. The protein suspensions were then digested with 40 µL of trypsin (Promega, Madison, WI, USA) buffer (6 µg trypsin in 40 µL of dissolution buffer) at 37 °C for 18 h. Finally, the filter unit was transferred to a new tube, and 40 µL of dissolution buffer was added followed by centrifugation at 14,000× g for 30 min. The resulting peptides were collected as a filtrate, and the peptide concentration was analyzed at OD280.

### Off-Line High-pH Reversed-Phase Fractionation

Nine serial samples from each subject and the common reference sample were included in one TMT10 labeling experiment set as shown in Fig. 1C. This labeling strategy avoids the potential for missing value issues resulting from data-dependent MS/MS acquisitions (Zimmer et al., 2006). TMT-labeled peptides were subjected to High-pH Reversed-Phase Fractionation in 1100 Series HPLC Value System (Agilent) equipped with a Gemini-NX (Phenomenex, 00F-4453-E0) column (4.6 × 150 mm, 3 µm, 110 Å) (Wang et al., 2011; Batth, Francavilla & Olsen, 2014). The peptides were eluted at a flow rate of 0.8 mL/min. Buffer A consisted of 10 mM ammonium acetate, pH 10.0, and Buffer B consisted of 10 mM ammonium acetate, 90% v/v acetonitrile (ACN), pH 10.0. Both buffers were filter sterilized. The gradient to perform the separation consisted of several steps: (1) 100% Buffer A for 40 min, 0%–5% Buffer B for 3 min; (2) 5%–35% Buffer B for 30 min; (3) 35%–70% Buffer B for 10 min; (4) 70%–75% Buffer B for 10 min; (5) 75%–100% Buffer B for 7 min; (6) 100% Buffer B for 15 min; and (7) 100% Buffer A for 15 min. The elution process was monitored by measuring absorbance at 214 nm, and fractions were collected every 1.25 min. The collected fractions (approximately 40) were finally combined into 10 pools. Each fraction was concentrated via vacuum centrifugation and reconstituted in 10 µL of 0.1% v/v formic acid. All samples were stored at −80 °C until LC-MS/MS analysis.

### Quantitative LC- MS/MS

The labeled samples were analyzed using the Easy-nLC nanoflow HPLC system connected to the Thermo Scientific™ Orbitrap Fusion™ Tribrid™ mass spectrometer (Thermo Fisher Scientific, San Jose, CA, USA). A total of 1 µg of each sample was loaded onto the Thermo Scientific EASY column (two columns) using an autosampler at a flow rate of 200 nL/min. Sequential separation of peptides on the Thermo Scientific EASY trap column (100 µm × 2 cm, 5 µm, 100 Å, C18) and analytical column (75 µm × 25 cm, 5 µm, 100 Å, C18) was accomplished using a segmented 60 min gradient from 5% to 28% Solvent B (0.1% formic acid in 100% ACN) for 40 min followed by 28%–90% Solvent B for 2 min and then 90% Solvent B for 18 min. The column was re-equilibrated to its initial highly aqueous solvent composition before each analysis. The mass spectrometer was operated in a positive ion mode, and MS spectra were acquired over a range of 375–1,500 m/z. The resolving powers of the MS and MS/MS scans at 200 m/z for the fusion were set as 120,000 and 50,000, respectively. The data-dependent mode was top speed, cycle time was 3 s, and ions were fragmented through high energy collisional dissociation. The maximum ion injection times were set at 50 ms for the survey scan and 105 ms for the MS/MS scans, and the automatic gain control target value was set to 4e5 for MS and to 1e5 for MS/MS. The dynamic exclusion duration was 40s.

### Database search

All raw files were analyzed using the Proteome Discoverer 2.1 software (Thermo Fisher Scientific). A search for fragmentation spectra was performed using the MASCOT search engine (Matrix Science, London, UK; Version 2.2) embedded in Proteome Discoverer against the Uniprot Canidae protein sequence database (released in November 2016, 31,596 sequences). Several search parameters were used: (1) monoisotopic mass; (2) trypsin as the cleavage enzyme; (3) two missed cleavages; (4) TMT labeling; (5) cysteine carbamidomethylation as fixed modifications; (6) peptide charges of 2+, 3+, and 4+; and (7) methionine oxidation. The mass tolerance was set to 20 ppm for precursor ions and to 0.1 Da for the fragment ions. Peptide spectral matches were filtered to a 1% false discovery rate. The relative quantitative analysis of the sample proteins was based on TMT reporter ion ratios from all unique peptides representing each protein. This analysis was performed using Proteome Discoverer (version 2.1). The relative peak intensities of the TMT reporter ions released in each of the MS/MS spectra were used. The final ratios obtained from the relative protein quantifications were normalized based on the median average protein quantification ratio. Only unique peptides obtained with a confidence percentage of >95% were included in the ratio ≥1.20, or ≤0.8333-fold cutoff value was used to identify upregulated and downregulated proteins with *p* < 0.05. The mass spectrometry proteomic data have been deposited to the ProteomeXchange Consortium via the PRIDE partner repository with the dataset identifier PXD lycorine (accession number PXD012936). Protein-protein interaction (PPI) analysis of DEP proteins STRING (Search Tool for the Retrieval of Interacting Genes/Proteins) version 11.0 was employed in this study for the potential PPI analysis of the DEPs proteins (http://string-db.org/) (Szklarczyk et al., 2015; Shannon et al., 2003). The parameter for confidence score was set to 0.4, and the yield PPI results were visualized by Cytoscape software (Shannon et al., 2003). The light blue nodes indicated upregulated-proteins and the light green nodes indicated downregulated-proteins. The edge thickness was proportional to the combined score of the proteins.

### Bioinformatics and enrichment analysis

Bioinformatics and enrichment analysis was performed as previously described (Hui Bin Huang, 2017).

### Western blot

Cells were homogenized on ice for 20 min in 1 × RIPA buffer with cocktail protease inhibitors (Roche) and then centrifuged at 15,000× g for 20 min at 4 °C. The supernatant was collected and then stored at −80 °C. For western blot analysis, equal amounts of proteins were separated using SDS-PAGE and subsequently transferred onto a polyvinyl fluoride membrane (Bio-Rad). The membrane was blocked with 5% BSA and then incubated with monoclonal antibody Nup93 (ab168805) and monoclonal antibody anti- β-actin (Cell Signaling Technologies) at 4 °C overnight. Membranes were washed in phosphate-buffered saline with Tween20 and then incubated for 2 h at RT with goat anti-rabbit horseradish peroxidase-conjugated secondary antibody (1:2,000; Cell Signaling Technologies).

### Statistical analyses

Statistical analyses were conducted as previously described (Hui Bin Huang, Zhang & Li, 2017).

## Results

### Conditions for lycorine treatment, viral infection, and group design

The concentration and exposure time of lycorine were determined to avoid any cytotoxic effects on the host cells and to ensure that the proteome of the host cells reflected the true response to the presence of lycorine. A prior study found through CCK-8 assay that 0.52 µM lycorine is not toxic to cells at 12 h post-infection (h.p.i.) (He et al., 2013). In addition, the time of lycorine addition was optimized. Results showed that the earlier lycorine was added, the better effect on viral titers. Therefore, a single replication cycle of influenza virus (12 h.p.i.) within lycorine treatment at 0.52 µM was chosen because it reflects a single viral replication and is a non-toxic concentration of lycorine at the time addition assay. Dose–effect relationship experiments showed that inhibition of lycorine of AIV becomes invalid within high-dose virus (Jun He et al., 2012; He et al., 2013). Hence, we ensured that the response of the whole cell proteome is caused by early infection and that the activity of lycorine is effective. Thus, 3 MOI was selected as the minimum infectious dose for a single cycle of viral infection for studying the single viral replication cycle (Peschel et al., 2013). Therefore, 3 MOI of AIV infections, lycorine concentration of 0.52 µM, and 12 h of incubation time were selected as the optimal dose and exposure time for further experiments.

In this study, we applied a TMT method, which has been widely applied for quantitative proteomic cell biology studies and many model organisms, to explore potent anti-viral agents and different signaling pathways (Lombardi et al., 2015; Burton et al., 2016). A schematic of the experimental workflow and Venn diagram of V/M, L/V, and L/M is shown in Fig. 1C. Each experiment group consisted of three biological replicates (*n* = 3). In total, 5,786 proteins were identified and relatively quantified at 0.01% FDR in the proteome using MaxQuant (Table S1). Among the statistically significant proteins detected by ANOVA (*p* < 0.05), protein abundances that changed <1.2-fold, >0.8333-fold, and *p* > 0.05 were discarded. The proteome profiles of mock control and infected cells were compared (V/M) to determine the effects of AIV infection on MDCK cells. Among those protein groups, 1101 proteins were identified, of which 340 DEPs were determined in the V/M group. The proteome profiles of the infected cells and lycorine-treated infected cells were compared to determine the effects of lycorine treatment on the infected cells. A total of 1059 proteins were identified in the V/L group, of which 258 were DEPs. These results indicated wide differences of protein expression in the HPAIV infection and lycorine treatment. Inoculating with HPAIV and lycorine resulted in 691 identified DEPs (76 + 86 + 42 + 54 + 189 + 159 + 85) among the three groups of MDCK cells in response to HPAIV infection and lycorine treatment, as shown in the Venn diagram (Fig. 1B).

### H5N1 AIV-induced cellular protein content changes in MDCK cells

**Figure 2:**
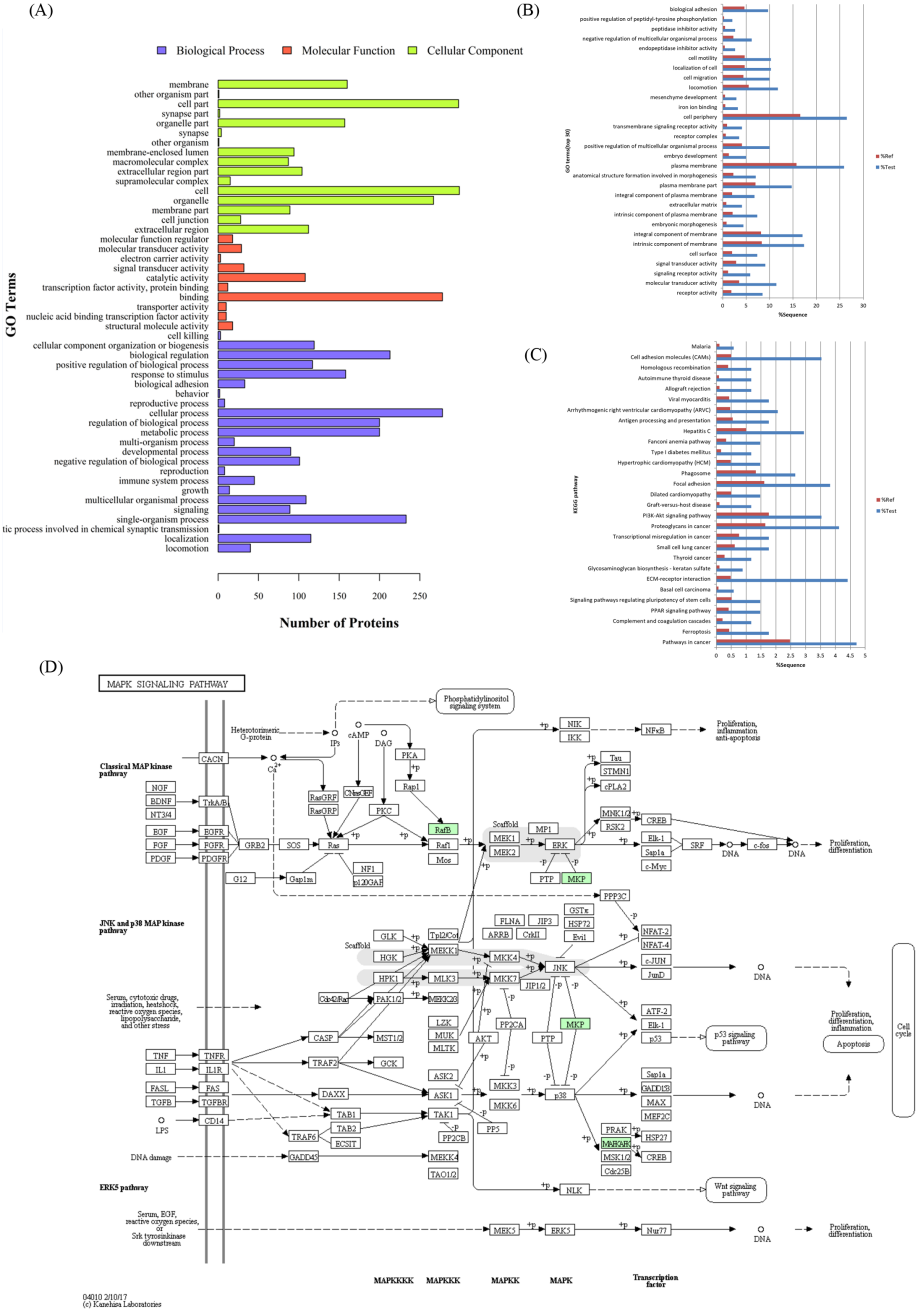
Differentially expressed proteins in the V/M groupinvolved in various biological processes, molecular functions, and cellularcomponents. (A) These proteins covered a wide range of biological processes, molecular functions, and cellular components, which were exhibited in different proportions in the V/M group. (B, C) Enrichment analysis of gene ontology terms and KEGG database pathways. (D) Representative viral replication-related pathway maps of differentially abundant proteins involved in the mitogen-activated protein kinase pathway in KEGG. The green colored proteins were identified using the tandem mass tag (TMT) approach.

The proteome profiles of the GD178-treated MDCK cells and mock control were compared (V/M) to determine the effects of GD178 influenza virus on MDCK cells. The V/M group contained 123 upregulated (>1.2-fold) and 217 downregulated (to <0.8333-fold) proteins in the V/M group (Table S2). Some of the proteins in the same network were direct interaction or through intermediate partner at the PPI level, as shown in Fig. S1. Gene ontology analyses of the proteins upregulated and downregulated by GD178 were performed to map the genes involved in different cellular processes, including biological and functional events (Fig. 2A). A large number of biological process GO terms were identified. The DEPs were strongly represented by “catabolism, metabolism, and physiological process”; the infected cells were also assigned to numerous molecular functions, of which “binding, catalytic and molecular transducer activities” were the main ones, and cellular components, of which “extracellular region part, organelle and membrane” were dominant. GO enrichment analysis was conducted on cell periphery (90 proteins), plasma membrane (88 proteins), intrinsic component of membrane (59 proteins) and molecular transducer activity (39 proteins) to further study the impact of DEPs in cell physiological processes and discover their internal relations (Fig. 2B). Several identified proteins have known interactions with one another and function in the same biochemical pathways. Therefore, a KEGG pathway-based enrichment analysis was applied to identify the main pathways that were potentially affected by the DEPs in the V/M group (Fig. 2C). The enriched pathways showed that the proteins were involved in pathway in cancer (path: ko05200), extracellular matrix (ECM)–receptor interaction (path: ko04512), focal adhesion (path: ko04510), mitogen-activated protein kinase signaling pathway (path: ko04010), and PI3K-Akt signaling pathway (path: ko04151). These data indicated that there are many changes in the protein profile in response to influenza virus infection at 12 h.p.i.

### Differentially expressed proteins in lycorine-treated and virus-infected cells

**Figure 3:**
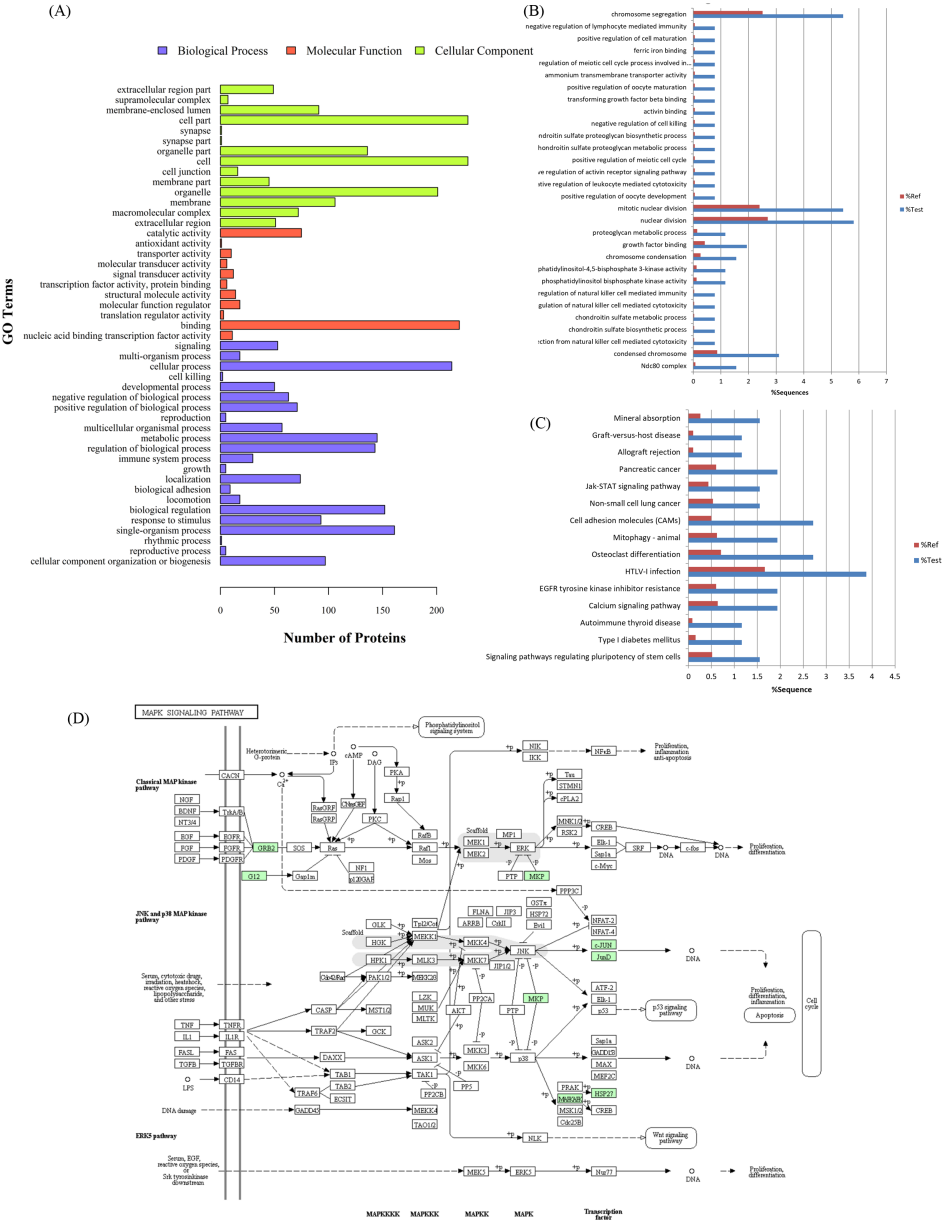
Differential proteins involved in various biological processes, molecular functions, and cellular components in the V/L group. (A) These proteins covered a wide range of biological processes, molecular functions, and cellular components, which were exhibited in different proportions in the V/L group. (B, C) Enrichment analysis of GO terms and KEGG pathway. (D) Representative lycorine response to viral replication-related pathway maps of differentially abundant proteins involved in the MAPK pathway in KEGG. Green colored proteins were identified using the TMT approach.

The proteome profiles of the infected cells and the infected cells within lycorine treatment were compared (V/L) to determine the effects of lycorine treatment on the infected cells. In total, 258 DEPs were found (Table S3). The PPI network was performed to determine whether these DEPs interact with each other and form protein complexes (Fig. S2). Two-hundred six proteins with at least 1.2-fold upregulation between the lycorine-treated and virus control group were found (V/L), and 52 proteins were significantly downregulated. The GO terms of DEPs were strongly represented by “cellular process, single-organism process, and biological regulation” in the biological process and “binding, catalytic and molecular transducer activities” in molecular functions; the lycorine-treated cells were also assigned to numerous cellular components, of which “membrane-enclosed lumen, organelle and membrane” were dominant (Fig. 3A). GO enrichment analysis showed that the three main parts were nuclear division (15 proteins), chromosome segregation (14 proteins), and condensed chromosome (eight proteins) (Fig. 3B). The functional classification of DEPs was conducted by KEGG enrichment analysis, and each protein was assigned to at least one of the following pathways: human T-lymphotropic virus-1 infection pathway (path: ko05166), cell adhesion molecules (CAMs) (path: ko04514), epidermal growth factor receptor tyrosine kinase inhibitor resistance (path: ko01521), Janus kinase-STAT signaling pathway (path: ko04630), and pancreatic cancer (path: ko05212) (Fig. 3C). These results showed differences between V/M and V/L, and further analyses of these genes that were co-changed in the two groups may shed light on the antiviral mechanism of lycorine.

### Characterization of proteins in the same trend in the V/M and V/L groups

**Figure 4:**
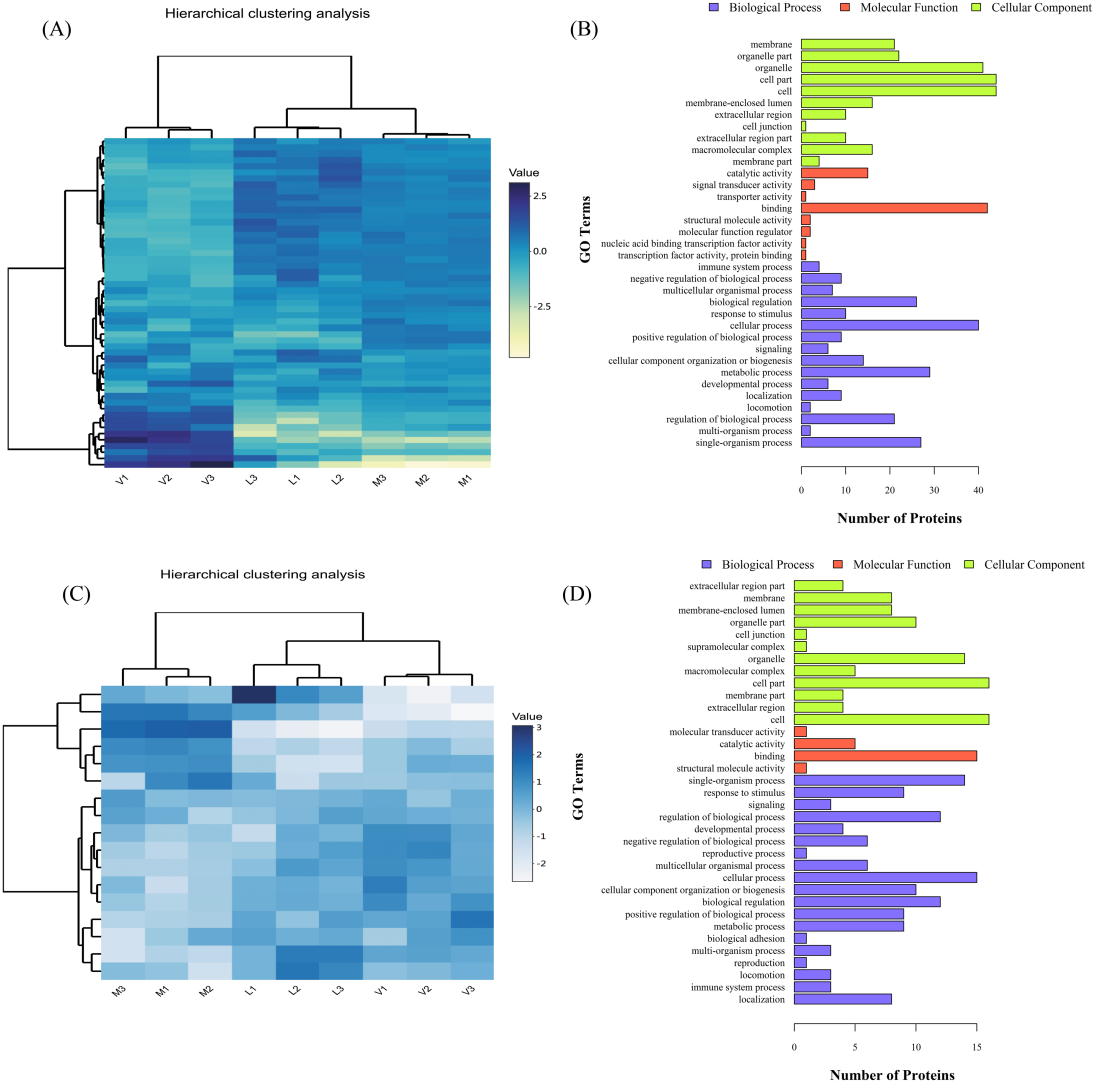
Effect of lycorine on viral infection cells. Expression levels of the 71 proteins in the V/M and V/L groups are shown. (A) Heat map of co-upregulated proteins in the two groups. Each quantile was separately analyzed for gene ontological pathways and clustered for z transformed p values. (B) Gene Ontology classification of DEPs in co-upregulated proteins of the two groups. (C) Heat map of co-downregulated proteins in the two groups. (D) Gene Ontology classification of DEPs in co-downregulated proteins of the two groups.

The proteins co-regulated in V/M and V/L were selected for further analysis. These 71 DEPs were investigated and annotated using GO terms and subjected to GO functional analysis (Table S4). Fifty-four proteins were upregulated in the V group but downregulated in the L group; this finding indicated that some of the proteins may participate in viral infections and may be blocked by lycorine to avoid progeny virus budding. Seventeen proteins were downregulated in the V group but induced in the L group, implying that these proteins were downregulated after influenza virus infection but increased after lycorine treatment. A total of 71 proteins showed upregulated or downregulated difference in abundance, and a heat map of these proteins was obtained (Figs. 4A and 4C). All co-upregulated and co-downregulated proteins were further analyzed by GO enrichment analysis, and each protein belonged to at least one term. Among the upregulated proteins, the three main terms in the BP category were cellular process (40 proteins), metabolic process (29 proteins), and organic substance metabolic process (29 proteins). The main terms in the MF category were transcription factor activity (42 proteins) and nucleic acid binding transcription factor activity (33 proteins). The main terms in the CC category were membrane part (49 proteins) and cell junction (41 proteins). In the downregulated proteins, the main terms in the BP category were localization (15 proteins), immune system process (14 proteins), and reproduction (12 proteins). The main terms in the MF category were structural molecule activity (15 proteins) and binding (11 proteins). The main terms in the CC category were extracellular region (16 proteins) and membrane part (16 proteins) (Figs. 4C and 4D). The analysis suggested that these biological functions may be affected by the treatment of AIV-infected MDCK cells with lycorine.

In addition, pathway analysis showed that these proteins were involved in viral replication-related pathways. For instance, oligoadenylate cyclase (Oasl1), serine/threonine protein kinase (STK10), and other proteins (8/71) were involved in adenosine and guanine triphosphatase (ATP and GTP) activity-related pathways; other pathways such as Nup93, mitochondrial translational initiation factor (MTIF3), and other proteins (9/71) participated in host cell responses to lycorine-related transcription or translation processes, such as cellular responses to stimulus and oxoacid-related metabolic processes. This may or may not be related to the antiviral properties of lycorine.

### Validation of protein profiles and Western blot of DEPs

**Figure 5:**
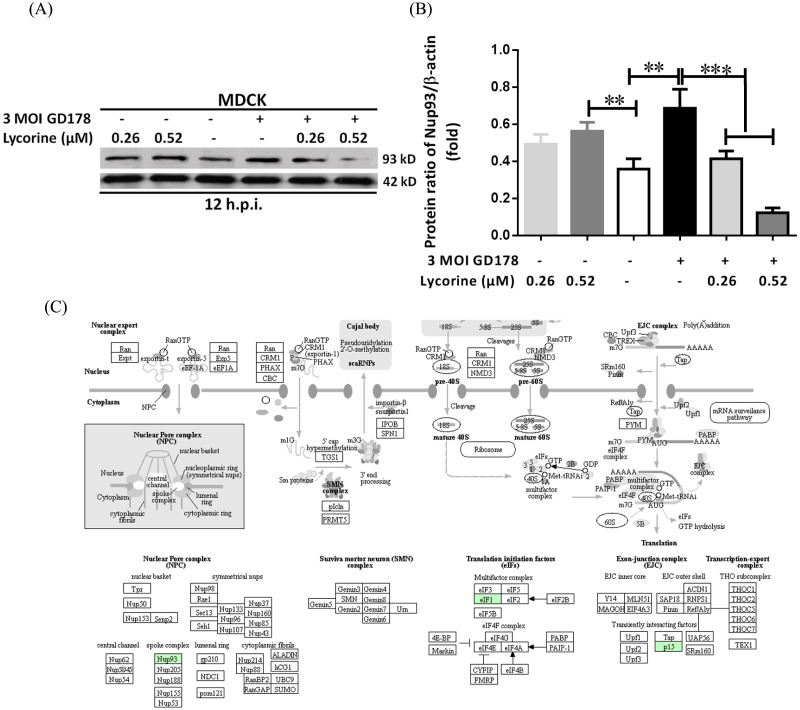
Western blot analysis demonstrating Nup93 inhibition by lycorine treatment in MDCK cells. (A) Lycorine was added at two concentrations (0.26 and 0.52 µM) to uninfected and infected cells until 12 h.p.i.. (B) Semi-quantification of proteins detected by western blot using ImageJ software. Values represent mean ± SD of two representative experiments. (C) Nup93-related pathway maps of differentially abundant proteins involved in RNA transport in KEGG. Green proteins were identified using the TMT approach.

According to our prior study, vRNP is retained in the nucleus under conditions of lycorine treatment to stop the cycle of influenza virus, which leads us to search for the proteins involved in the vRNP export pathway (He et al., 2013). For several pathways relevant to proteins targeting nuclear export were enriched in the “biological process” category. The proteins with the same trend in the V/M and V/L groups were our candidate proteins. Fortunately, a gene directly related to nuclear transport, *Nup93*, was upregulated by 1.29-fold, in contrast to the low expression level (0.8) found in lycorine treatment over controls (Table S2). From the GO terms, Nup93 is related with export of material from the nucleus, such as mRNA, tRNA, viral material, and viral transcription (Table S4). Given its possible role in viral inhibition (Muhlbauer et al., 2015), western blotting assay with specific antibodies was performed to determine if the expression regulation for these proteins is at the protein level. As shown in Fig. 5, the trends in Nup93 level changes in the infected cells and lycorine treatment groups were similar to the changed patterns as was observed. Moreover, the protein levels of Nup93 in the uninfected cells but treated with the same concentrations of lycorine were not changed as such as the cells treated with lycorine after influenza virus infection. These results indicate that Nup93 expression was induced after influenza virus infection but was dramatically decreased after lycorine treatment at 0.52 µM. This concentration did not exhibit toxicity to the uninfected cells, which was in accordance with the TMT results. As a result, AIV infection may induce Nup93 to complete the viral cycle, and the process of protein targeting into Nup93 after lycorine treatment may partly be explained by the blockage of vRNPs in the host cellular nucleus.

## Discussion

An effective drug for HPAIV treatment is still a worldwide problem (Arai et al., 2018). Our previous study found that lycorine exhibits stronger anti-influenza activity against GD178 than oseltamivir and retains vRNP in the nucleus (He et al., 2013). Hence, a comprehensive description of changes in viral and cellular proteins during AIV infection and lycorine treatment based on multiplex TMT-based quantitation was carried out. The interaction of the host cellular proteins with lycorine and the factor of nucleus transport are two of our main research interests.

Proteomics is the large-scale study of molecules known as proteins, the critical building blocks of both host and viruses (Drayman et al., 2017; Kim et al., 2017; Kleiner et al., 2017; Vasilijevic et al., 2017). Influenza virus modifies and hijacks numerous processes and cell organelles during its replication cycle (De Castro Martin et al., 2017); it also regulates the subcellular localization of protein complexes. In the present study, these phenomena were observed in the HPAIV-infected results (Fig. 2 and Table S2) in conjunction with the total number of significantly regulated proteins (340 proteins). On the basis of the numbers of proteins being significantly modulated and the pathways associated with those proteins, the GD178 influenza virus induced more profound responses to ECM–receptor interaction, focal adhesion, and PI3K–Akt signaling pathway through KEGG pathway-based enrichment analysis. Yek et al. (2016) showed that the major function modulated by myxovirus (influenza virus) resistance 1 infection is predominantly enriched in the ECM–receptor interaction and focal adhesion pathways. Liu et al. (2012) showed that infection with a H5N1 HPAIV strain with an MOI of 0.5 cannot infect much of the cells in their study. Thus, 3 MOI or higher infection doses are better for studying one single viral replication cycle. Considering the dose effects and efficiency of lycorine and GD178 infection, we chose 3 MOI for the single cycle. Therefore, a suitable time point (12 h.p.) must be selected to harvest virus-infected cells that have been treated by lycorine and can be used for proteomic analysis.

After endocytic cell entry, vRNPs are released into the cytoplasm and enter the nucleus where viral mRNA synthesis and replication occur. The viral genomes are encapsulated with nucleoproteins (NPs), and they are associated with trimeric polymerase complexes (the viral proteins M1, NEP/NS2, and NP) and recognized by CRM1 to the cytoplasm via the NPC (Flatt & Greber, 2015). NPC is a highly conserved protein complex that is localized at the nuclear periphery and required for the import and export of proteins and RNA (Labade, Karmodiya & Sengupta, 2016). The influenza virus could induce proteins and pathways that it uses on dependently, replicate its genome in the nucleus, and export it to the cytoplasm via NPCs. The nuclear pores promote the nuclear export of newly synthesized RNPs, which is an important process that regulates cellular functions and facilitates viral NPC assembly. Muhlbauer et al. (2015) proved that virus-induced cellular caspase activities cause a widening of nuclear pores, thereby facilitating nucleocytoplasmic translocation. However, they focused on another NPC protein, Nup153, which is correlated with the import and export pathway for influenza virus infection. In the present study, the expression of Nup93 was upregulated by 1.29-fold after GD178 infection at 12 h. However, the different NPC proteins and different functions render direct comparison with our results impossible. The MDCK cells infected with GD178 exhibited tremendous changes in the levels of many proteins and pathways. Interestingly, the NPC protein Nup93 was confirmed to be directly related to the export of mRNA/tRNA from the nucleus. We will pay close attention to the changes in Nup93 expression after lycorine treatment.

Lycorine, the main constituent from *Lycoris radiata* bulbs, exhibits a wide range of biological activities, including antiviral (Masi et al., 2016), antimalarial (Cho et al., 2018), antibacterial (Bendaif et al., 2018), anti-parasitic and anti-inflammatory (Park, 2014). The first reported activity of lycorine is the inhibition of the termination of protein synthesis in poliovirus infection (Vrijsen et al., 1986). Subsequent studies found that lycorine exhibits antiviral activity toward herpes simplex virus (Renard-Nozaki et al., 1989), HIV-1 (Lin et al., 1995), coronavirus (Li et al., 2005), poliovirus (Hwang et al., 2008), West Nile Virus, dengue and yellow fever viruses (Zou et al., 2009), enterovirus 71 (Liu et al., 2011), influenza virus (He et al., 2013), hepatitis C virus (Guo et al., 2016), and adult zika virus (Masi et al., 2016). Although lycorine is a compound with various antiviral activities, the molecular mechanism underlying the effects of lycorine is still unclear.

Compared with other pharmacological activity mechanisms, studies on anti-cancer activity have gained deep insights (Lamoral-Theys et al., 2010). Potential targets for lycorine action include Bcl-2 family proteins Bcl-2 and Mcl-1, HDAC, TNF- *α*, STAT, and HMGB1. However, no specific target for lycorine-induced anticancer effect has been identified so far. In the present study, it is most obvious by examination of lycorine treatment after HPAIV-infected results (Fig. 3 and Table S3). On the basis of 258 proteins being significantly modulated and the pathways associated with those proteins, the lycorine-treated cells induced more profound responses to CAMs, EGFR-related pathway, and JAK-STAT signaling pathway through KEGG pathway-based enrichment analysis. Shen et al. authenticated that lycorine directly interacts with EGFR and inhibits EGFR activation (Shen et al., 2018). Hu et al. (2015) and Jin et al. (2016) showed that lycorine inactivates the JAK-STAT signaling pathway to inhibit the proliferation of cancer cells. Furthermore, our present results agree with these specific signaling pathways. In addition, GO enrichment analysis showed that 15 proteins involved in nuclear division were differently expressed upon lycorine administration. Notably, Nup93 expression was decreased upon lycorine treatment.

The 71 DEPs that were co-upregulated or co-downregulated in both V/M and V/L groups were selected as candidates (Fig. 4 and Table S4). Among them, 54 candidate proteins were increased by GD178 infection but decreased by lycorine treatment. Viral infection played a significant down-modulatory role to the 17 candidate proteins that were upregulated by lycorine. However, the DEPs detected in the current study hardly match those determined by SILAC analysis conducted in 2014 and published in 2017 (Hui Bin Huang, 2017) by our group. This result might be due to the different instruments applied and databases used. The mass spectrometer Q-Exactive was applied for SILAC experiment in the previous study, whereas fusion-lumos instrument was used in the present study. At present, we found that Nup 93 protein was inhibited by lycorine treatment, which aroused our great interest. The same topic will be the focus of our follow-up work. To explore how lycorine affects nucleus transport, we analyzed the protein levels of Nup93 by Western blot assay and found that Nup93 levels were increased after HPAIV infection but lowered with lycorine treatment. Additionally, Nup93 had the same levels in both lycorine control group and mock group. Lycorine significantly reduced Nup93 expression after influenza virus infection, which may have affected nucleocytoplasmic transport instead of lycorine toxicity in the host cells.

NPCs are composed of approximately 30 proteins known as nucleoporins. Nup93 is one of the major subcomplexes of the NPC and is responsible for the correct assembly of the NPC (Sachdev et al., 2012). Given its time-of-addition effect, lycorine is involved in the early steps of influenza virus replication aside from virus binding and viral RNP activity (He et al., 2013). Nucleocytoplasmic transport is integral to the majority of the influenza virus explicative cycle and critical for the efficient replication of influenza virus. Importantly, shuttling of specific proteins out of the nucleus is essential for the regulation of the basic functions of the host cells. The functions of nucleo-cytoplasmic transport in regulating tumor growth, cell cycle, and apoptosis have become the therapeutic target of cancer (Gravina et al., 2014; Hill et al., 2014; Rosebeck et al., 2016). Recent data have indicated that viral genome transport through the NPC is not always smooth sailing but can be blocked. This result may be the basis for host defense mechanisms that evoke an intrinsic antiviral response (Flatt & Greber, 2015; Kirli et al., 2015). Furusawa, Yamada & Kawaoka (2018) showed that viral RNA accumulates in the nucleus of Nup93-depleted cells. This observation suggests that Nup93 is involved in the nuclear export of viral RNA of the viral life cycle. A deeper understanding of how AIV RNA is exported from the nucleus via NPCs will thus help in the development of new antiviral drugs.

## Conclusion

Lycorine is an inhibitor of influenza virus that effectively inhibits AIV infection. Comparative proteomic analysis revealed that treatment with lycorine alters the expression of a number of proteins in AIV-infected cells. This finding suggests that lycorine affects the protein expression in AIV-infected cells. Among the 71 DEPs that were modulated in both V/C and V/L groups, candidate targets were predicted to collectively inhibit viral infection (pathway analysis). GO and KEGG pathway analyses revealed that Nup93, as an important NPC component that directs nucleocytoplasmic transport, was induced by virus infection but sharply decreased by lycorine treatment. Therefore, Nup93 may serve as a potential antiviral target for genetic manipulation. However, further functional characterization of Nup93 is necessary before this possibility can be confirmed. For instance, Nup93 knockdown or knockout experiments may be performed on these proteins individually to evaluate the functional effects on AIV replication.

##  Supplemental Information

10.7717/peerj.7697/supp-1 Supplemental Information 1 PPI networks of GD178 infection (V group) significant proteins A: PPI network of V/M group

10.7717/peerj.7697/supp-2 Supplemental Information 2 dPPI networks of GD178 infection (V group) significant proteins B: dPPI network of V/M group

10.7717/peerj.7697/supp-3 Supplemental Information 3 PPIof lycorine treatment after viral infection A: PPI network of V/L group.

10.7717/peerj.7697/supp-4 Supplemental Information 4 dPPI network of V/L group. B. dPPI network of V/L group.

10.7717/peerj.7697/supp-5 Table S1 5,786 proteins were identified through these groups 5786 proteins were identified and relatively quantified at 0.01% FDR in the proteome using MaxQuant.

10.7717/peerj.7697/supp-6 Table S2 340 cellular proteins were differentially expressed proteins as shown by MaxQuant in the V/M group 340 cellular proteins were differentially expressed proteins as shown by MaxQuant in the V/M group, including 123 up-regulated (>1.2-fold) and 217 down-regulated (to <0.833-fold) proteins.

10.7717/peerj.7697/supp-7 Table S3 258 proteins were differentially expressed as shown by MaxQuant in the L/V group 258 proteins were differentially expressed as shown by MaxQuant in the M/H group, including 206 up-regulated (>1.2-fold) and 52 down-regulated (to <0.833-fold) proteins.

10.7717/peerj.7697/supp-8 Table S4 V/M vs V/L co-regulated proteins Seventy proteins have been identified in both co-upregulated and co-downregulated in V/M and V/L groups.

10.7717/peerj.7697/supp-9 Supplemental Information 9 Full-length uncropped blots for Figures 1 and 5 The samples from left to right were virus, virus, lycorine control (0.26 µM), lycorine control (0.52 µM), mock, lycorine treatment (0.26 µM) after virus infection, lycorine treatment (0.52 µM) after virus infection. The top row of protein is nup93. The bottom row is *β*-actin.

## Additional Information and Declarations

### Data Availability

The following information was supplied regarding data availability: Raw data is available at PXD (iProX): http://proteomecentral.proteomexchange.org/cgi/GetDataset?ID=PXD012936.
