# Supplementary figures and images for: Tandem mass tag-based quantitative proteomic analysis of lycorine treatment in highly pathogenic avian influenza H5N1 virus infection
Source: PeerJ. 2019 Oct 2;7:e7697. doi: 10.7717/peerj.7697 (PMC6778435; doi:10.7717/peerj.7697)

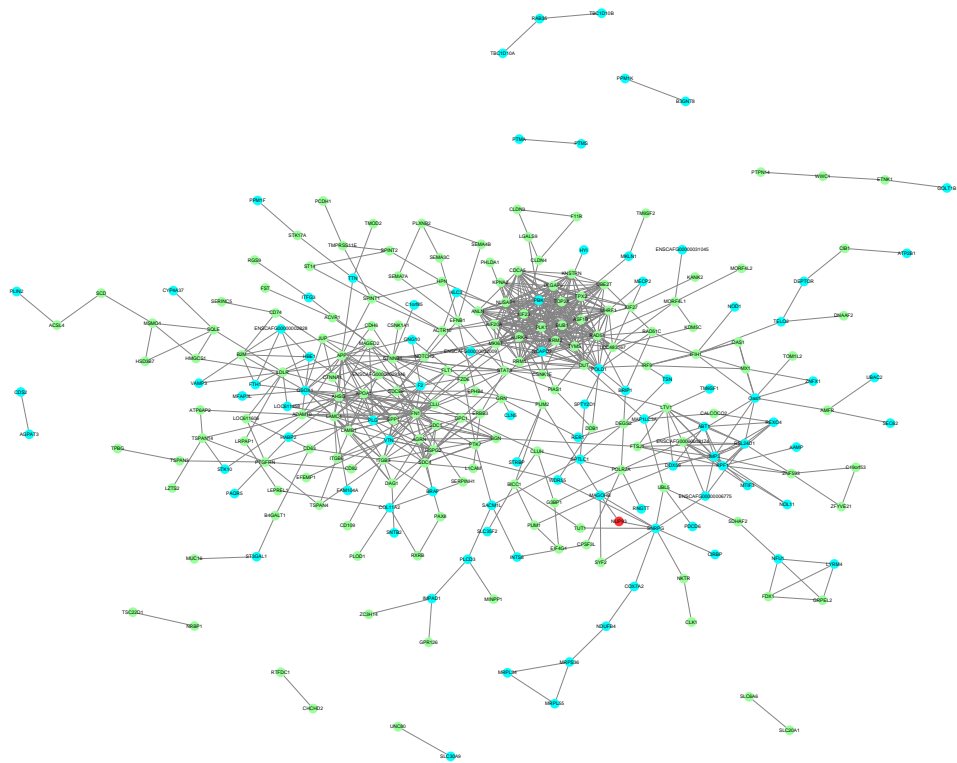

Supplement: Supplemental Information 1 — A: PPI network of V/M group [file peerj-07-7697-s001.pdf]

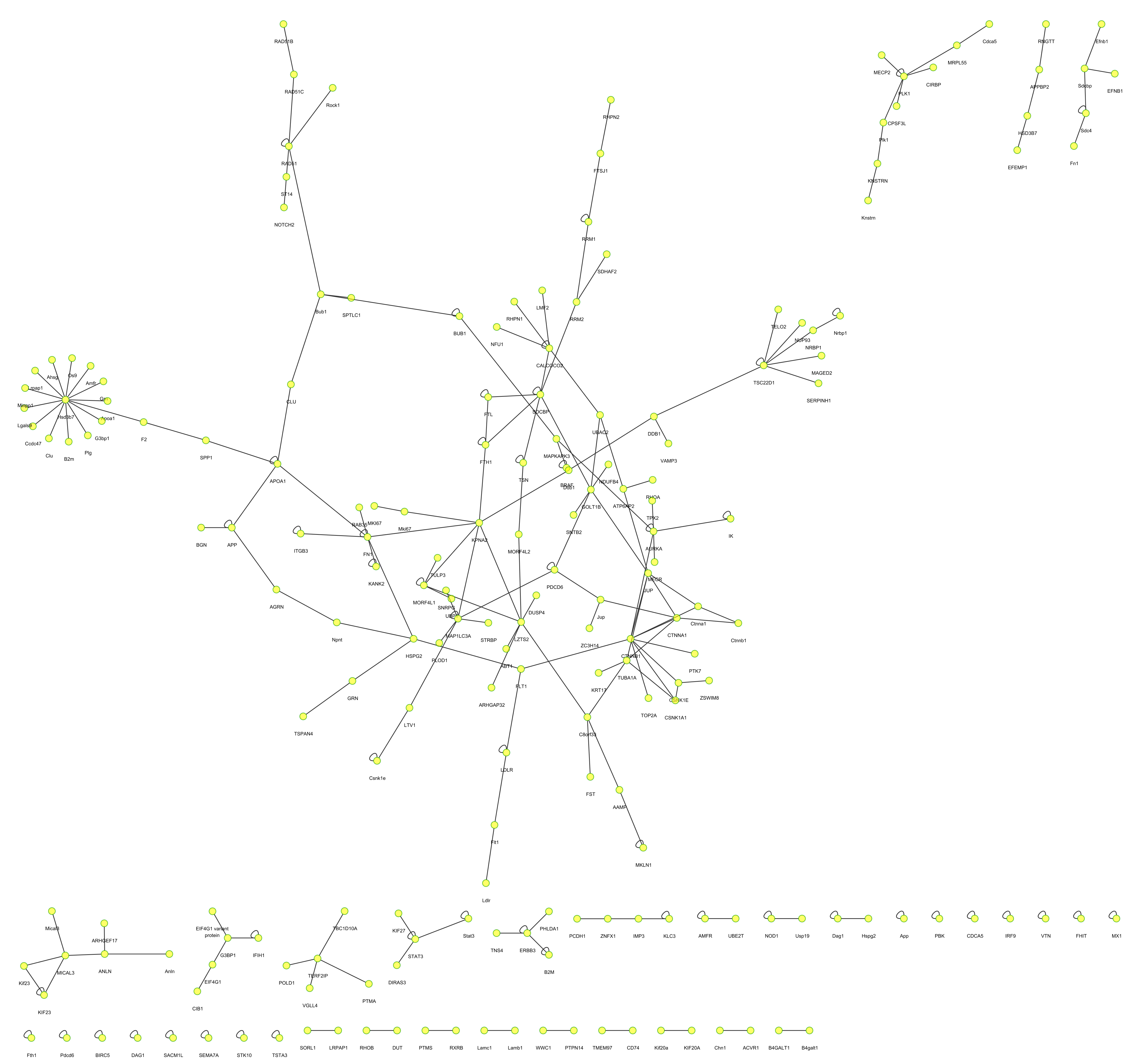

Supplement: Supplemental Information 2 — B: dPPI network of V/M group [file peerj-07-7697-s002.jpg]

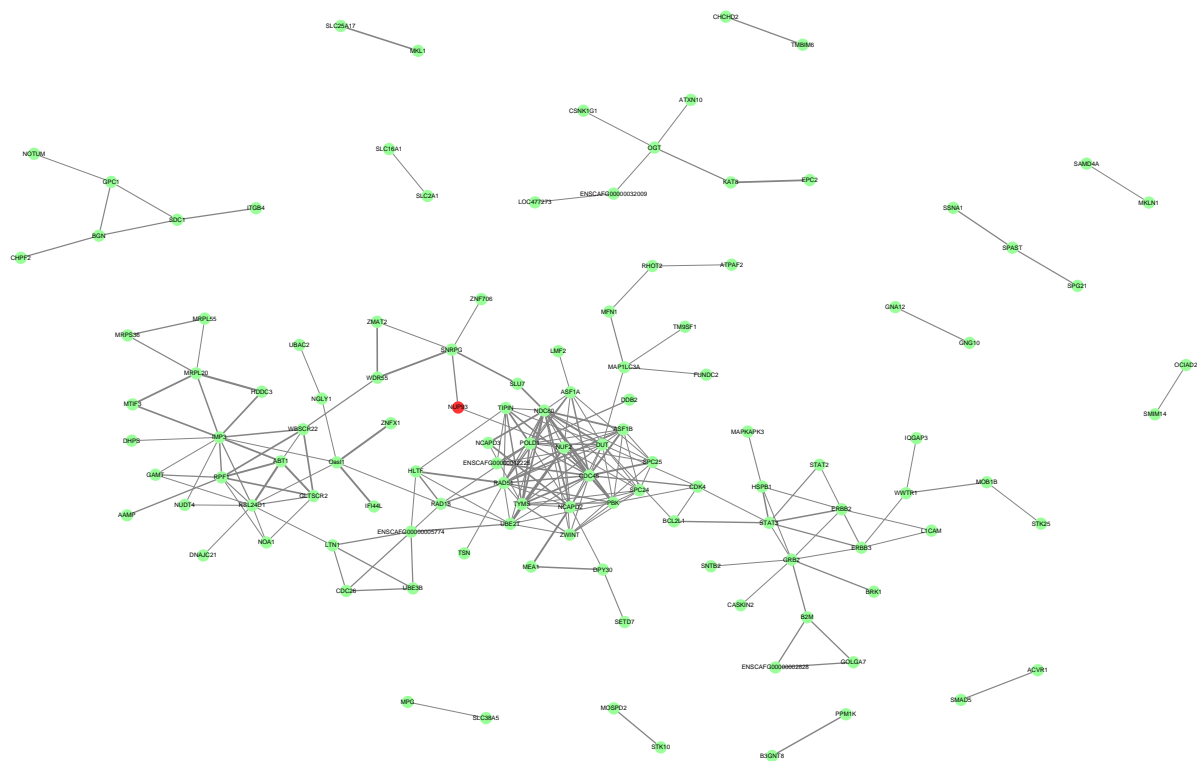

Supplement: Supplemental Information 3 — A: PPI network of V/L group. [file peerj-07-7697-s003.pdf]

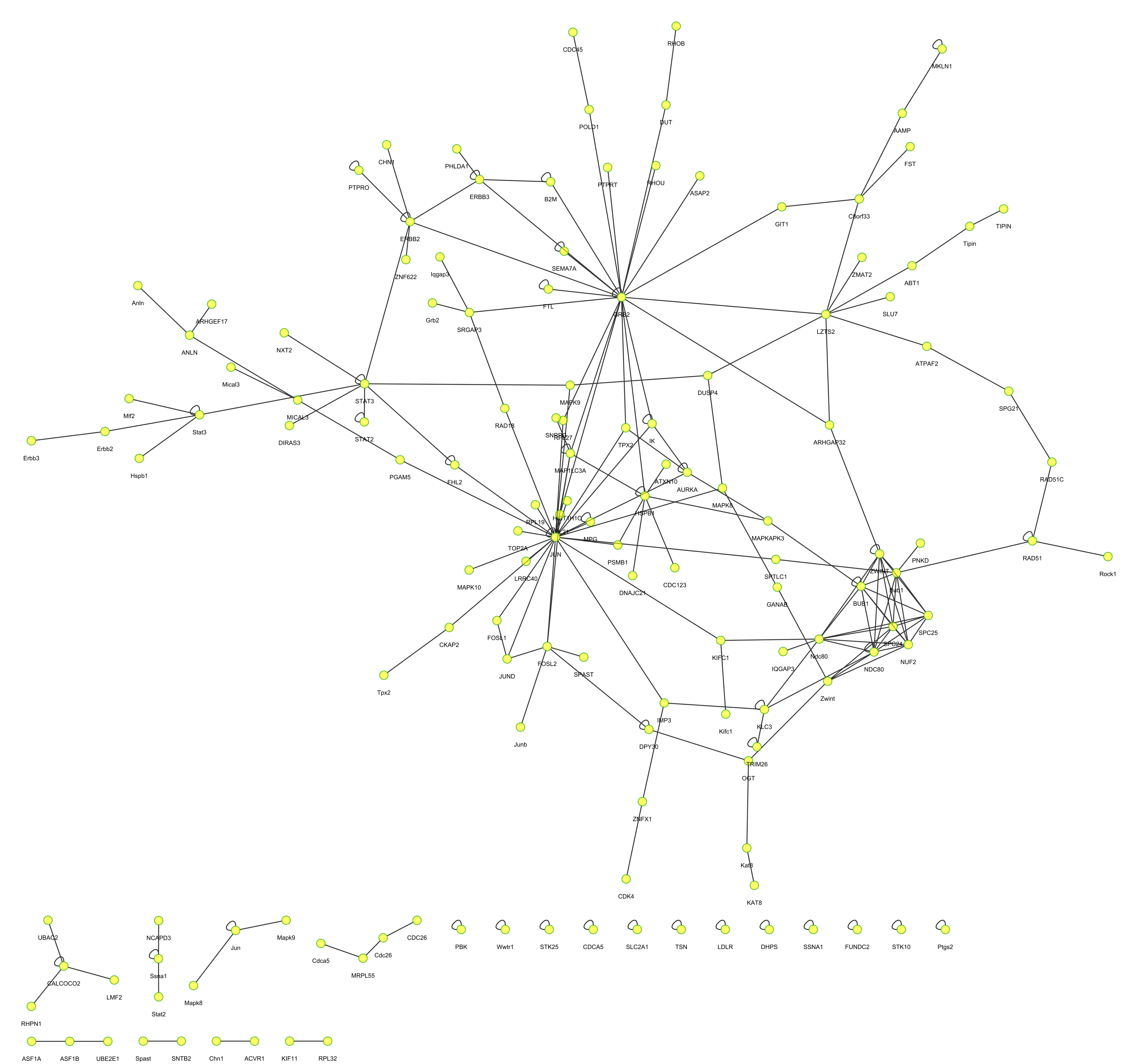

Supplement: Supplemental Information 4 — B. dPPI network of V/L group. [file peerj-07-7697-s004.jpg]

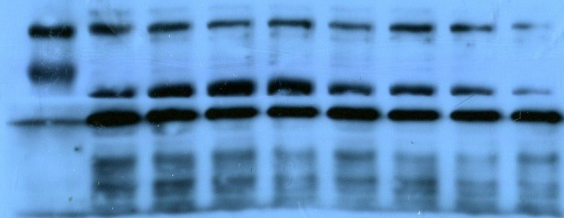

*B-actin*

Supplement: Supplemental Information 9 — The samples from left to right were virus, virus, lycorine control (0.26 µM), lycorine control (0.52 µM), mock, lycorine treatment (0.26 µM) after virus infection, lycorine treatment (0.52 µM) after virus infection. The top row of protein is nup93. The bottom row is β-actin. [file peerj-07-7697-s009.pdf]
